# Supplementary material for: Improving Atorvastatin Release from Polyelectrolyte Complex-Based Hydrogels Using Freeze-Drying: Formulation and Pharmaceutical Assessment of a Novel Delivery System for Oral Candidiasis Treatment
Source: Int J Mol Sci. 2025 Mar 4;26(5):2267. doi: 10.3390/ijms26052267 (PMC11900555; doi:10.3390/ijms26052267)
Supplement: Supplementary file 1 [file ijms-26-02267-s001.zip › ijms-3460666-supplementary.pdf]

Supplementary Materials

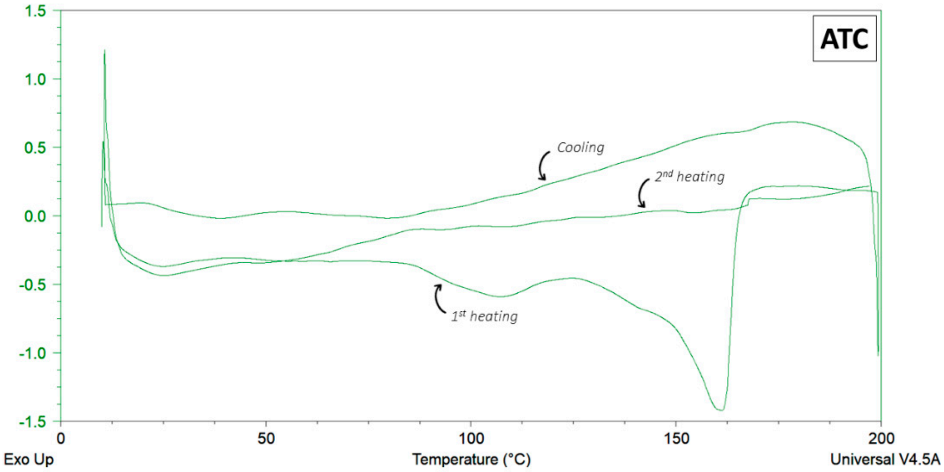

Figure S1. *DSC curves for pure atorvastatin calcium (ATC).*

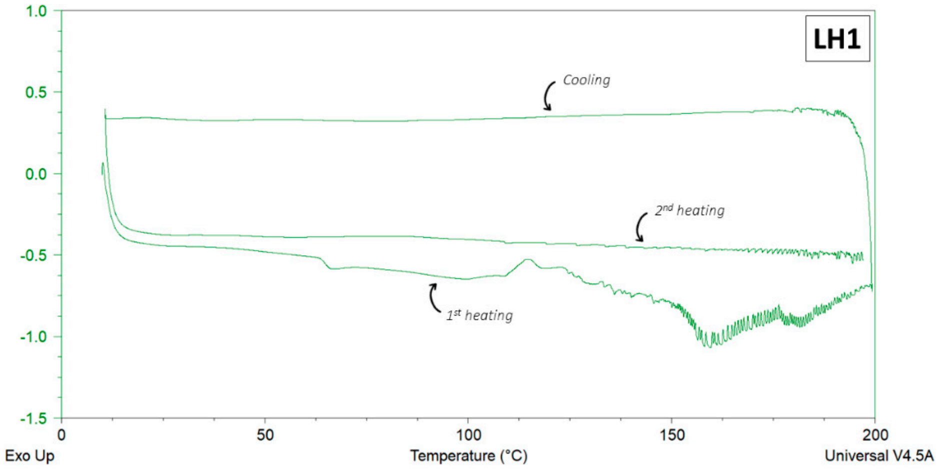

Figure S2. *DSC curves for LH1.*

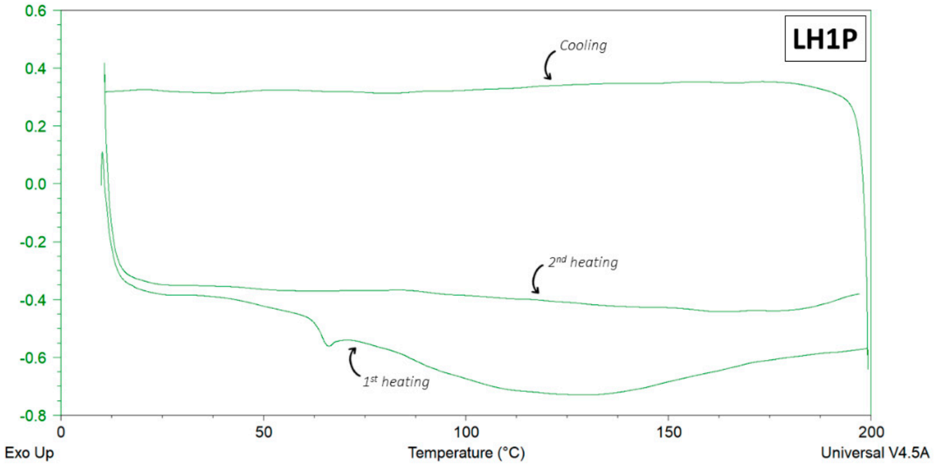

Figure S3. *DSC curves for the placebo LH1P.*

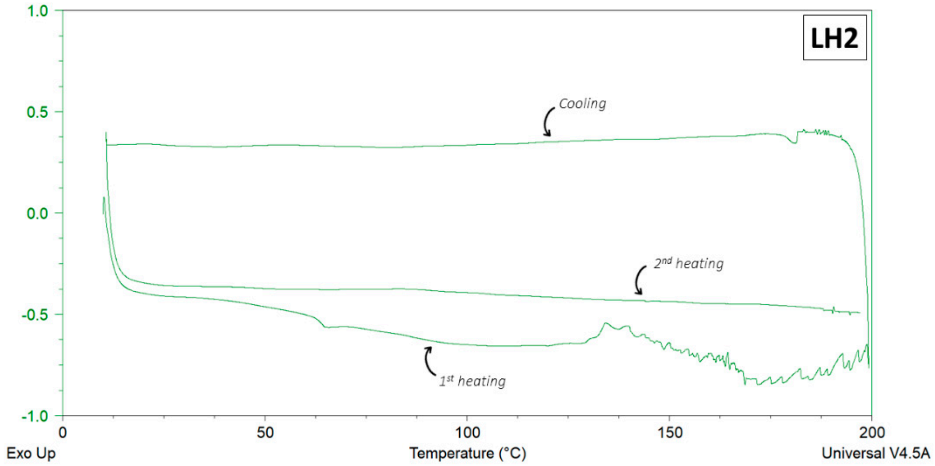

Figure S4. *DSC curves for LH2.*

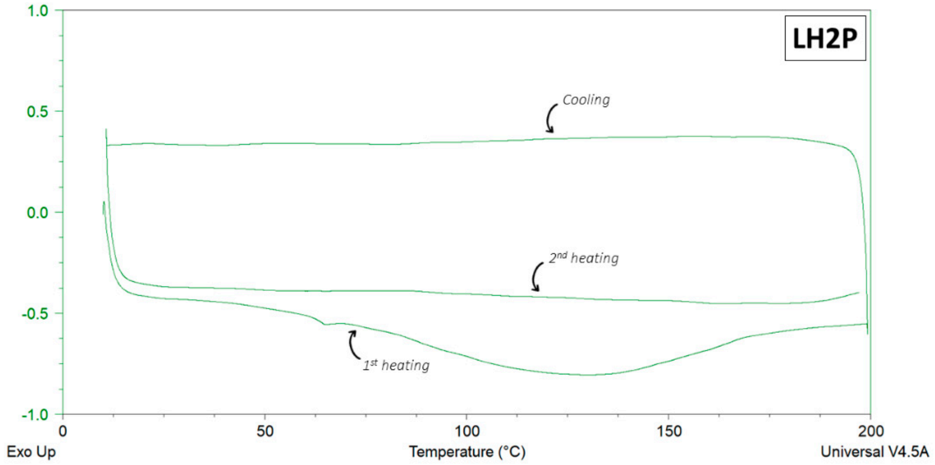

Figure S5. *DSC curves for the placebo LH2P.*

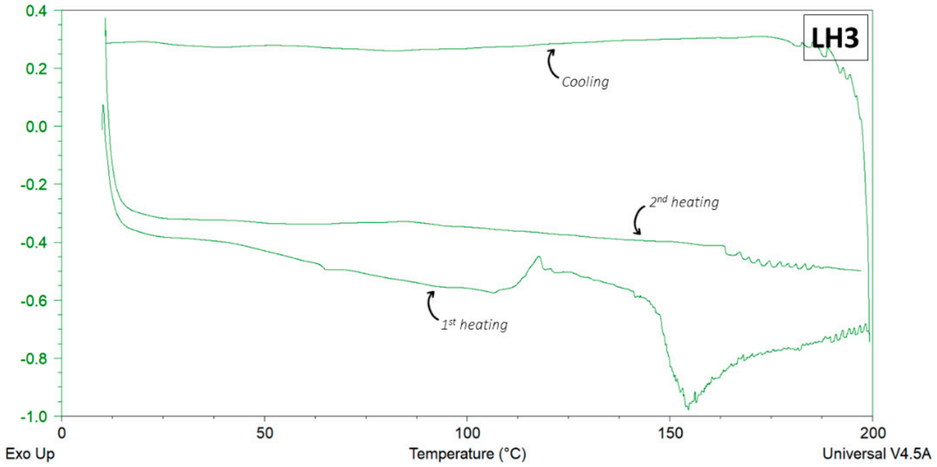

Figure S6. *DSC curves for LH3.*

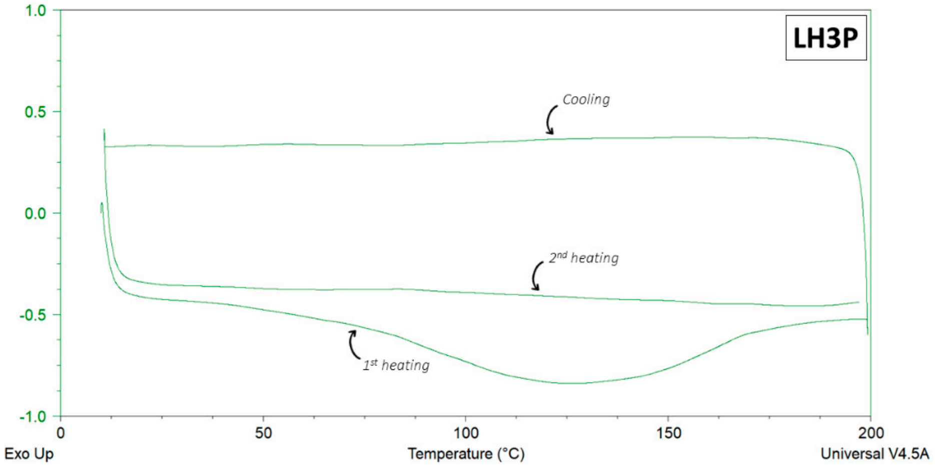

Figure S7. *DSC curves for the placebo LH3P.*
